# Expression of SARS‐CoV‐2 entry‐associated proteins in COPD airways: an immunohistochemical study

**DOI:** 10.1002/path.6477

**Published:** 2025-10-06

**Authors:** Larissa E Vlaming‐van Eijk, Zarlasht Sarsam, Janna Bakker, Marjan Reinders‐Luinge, Corry‐Anke Brandsma, Wim Timens

**Affiliations:** ^1^ Department of Pathology and Medical Biology University of Groningen, University Medical Center Groningen Groningen The Netherlands; ^2^ Groningen Research Institute for Asthma and COPD (GRIAC) University Medical Center Groningen Groningen The Netherlands

**Keywords:** COPD, SARS‐CoV‐2, COVID‐19, viral entry factors, immunohistochemistry, bronchial epithelium, protein expression, smoking

## Abstract

Coronavirus disease 2019 (COVID‐19) is of special concern to patients with chronic obstructive pulmonary disease (COPD), given their susceptibility to exacerbations caused by respiratory tract infections. As the susceptibility of acquiring a SARS‐CoV‐2 infection in COPD remains unclear, this study explored the airway expression of SARS‐CoV‐2 entry‐associated proteins in the lungs of COPD patients in comparison to non‐COPD controls. Immunohistochemical staining of lung tissue was performed to investigate the expression profiles of SARS‐CoV‐2 entry‐associated proteins in the bronchial epithelium of 27 COPD patients and 40 non‐COPD controls. In addition, the associations between these expression profiles with lung function in COPD patients and smoking status in non‐COPD controls were examined. COPD patients demonstrated smoking‐independent lower expression of HSPA5, NRP1, BSG, TMPRSS2, and ITGB6 in airway epithelium as compared to non‐COPD controls. No significant differences were observed for Furin, CTSL, ADAM17, and ITGA5. BSG percentage area expression was significantly negatively associated with lung function in COPD patients. Moreover, the study revealed smoking‐associated differences for Furin, HSPA5, ADAM17, BSG, ITGA5, and ITGB6 within non‐COPD controls, with lower airway epithelial expression (except for Furin) in ever‐smokers than in never‐smokers. To conclude, this study showed a lower expression of a specific set of SARS‐CoV‐2 entry‐associated proteins in the bronchial epithelium of COPD patients compared with non‐COPD controls, while other factors showed similar expression levels. The consequences of these findings on COVID‐19 susceptibility remain uncertain. Although reduced expression of entry factors may suggest less cellular availability for viral entry, it could be speculated that the similar expression levels of other factors, together with impaired airway clearance in COPD, may still facilitate infection, thereby providing potential mechanistic insight into COVID‐19 susceptibility in this patient population. © 2025 The Author(s). *The Journal of Pathology* published by John Wiley & Sons Ltd on behalf of The Pathological Society of Great Britain and Ireland.

## Introduction

Coronavirus disease 2019 (COVID‐19), caused by SARS coronavirus 2 (SARS‐CoV‐2), has disproportionately affected individuals with chronic obstructive pulmonary disease (COPD) [1, 2, 3, 4, 5, 6]. COPD is a common lung disease characterized by chronic respiratory symptoms caused by airway (chronic bronchitis) and alveolar (emphysema) changes in the lung, resulting in irreversible airflow limitation [7]. Prolonged exposure to toxic particles, including cigarette smoke, is a key risk factor in the pathogenesis of COPD, resulting in a decline in lung function, as demonstrated by the measurements forced expiratory volume in 1 s (FEV_1_) and FEV_1_/forced vital capacity (FVC) [7]. The Global Initiative for Chronic Obstructive Lung Diseases (GOLD) has classified COPD into four severity groups using a combined assessment based on lung function, current symptoms, and exacerbation risk [7].

Patients with COPD are particularly susceptible to respiratory exacerbations caused by viral respiratory tract infections [8]. Although COPD has been established as a significant risk factor for progression to severe illness in COVID‐19 [1, 2, 3, 4, 5, 6], the risk of acquiring SARS‐CoV‐2 infection remains unclear [9]. In addition, the true biological susceptibility might be underestimated, as COPD patients were more likely to adhere to infection control measures during the pandemic due to the concerns about increased COVID‐19 risk. A key component determining their biological susceptibility to COVID‐19 may involve the expression of factors that facilitate SARS‐CoV‐2 cell entry. Angiotensin‐converting enzyme 2 (ACE2) is the primary entry receptor for SARS‐CoV‐2 [10]. However, the tissue distribution of ACE2 does not fully correspond to the pattern of organ involvement observed in COVID‐19, as its expression in lung tissue is relatively low. This discrepancy has led to the hypothesis that alternative entry‐associated factors may contribute to viral entry in the lungs.

SARS‐CoV‐2 infection relies on the binding of its spike (S) protein to ACE2, after which the virus may enter the host cell either by direct fusion of the viral envelope to the host cell membrane (i.e. ‘cell surface entry’) or by internalization of the virus‐ACE2 complex through clathrin‐mediated endocytosis into endolysosomes (i.e. ‘endosomal entry’) [10, 11]. Both entry routes result in membrane fusion, which requires proteolytic activation of the S protein through sequential cleavage steps at the S1–S2 boundary—which is cleaved by Furin in the virus‐producer cell—and the S2’ site in the S2 subunit [10]. Depending on the route of SARS‐CoV‐2 entry, the S2’ site is cleaved by different proteases in the target cell, such as transmembrane protease serine 2 (TMPRSS2), cathepsin L (CTSL), and a disintegrin and metalloprotease 17 (ADAM17) [10, 12]. Alternative proteins, including neuropilin 1 (NRP1), basigin (BSG), and heat shock protein A5 (HSPA5), have also been suggested to serve as cofactors for SARS‐CoV‐2 entry [10, 13]. Lastly, integrins, such as ITGA5 and ITGB6, have been proposed to facilitate SARS‐CoV‐2 cell entry by interacting with the S protein, either through ACE2‐mediated entry or independently of ACE2 [14, 15, 16, 17].

The primary objective of this study was to investigate whether COPD patients exhibit altered expression patterns of alternative proteins involved in SARS‐CoV‐2 entry in their bronchial epithelium, as this could potentially indicate a risk of infection in COPD patients. Given that smoking is a major risk factor for COPD, we also examined the relationship between the airway epithelial expression patterns and smoking status. To this end, we used immunohistochemistry to compare SARS‐CoV‐2 entry‐associated protein expression in the bronchial epithelium between COPD patients and non‐COPD controls, while additionally examining the relationship between these airway expression patterns and smoking status as well as with FEV_1_% predicted (pred) as a measure of lung function and an indication of COPD severity.

## Materials and methods

### Ethics approval

This study was conducted in compliance with the Research Code of the University Medical Centre Groningen (UMCG), as outlined in https://umcgresearch.org/w/research-code-umcg, and adhered to national ethical and professional guidelines Code of Conduct for Health Research (https://www.coreon.org/wp‐content/uploads/2023/06/Code‐of‐Conduct‐for‐Health‐Research‐2022.pdf). The use of data and leftover lung tissue in this study was exempt from the Medical Research Human Subjects Act in The Netherlands, as confirmed by the Medical Ethical Committee of the UMCG. The study protocol was approved by the Central Ethics Review Board (study no. 202000687) and under Dutch laws (Medical Treatment Agreement Act [WGBO] art. 458; GDPR art. 9; UAVG art. 24) was exempt from the requirement for informed consent. All donor material and clinical data were deidentified before experimental use, ensuring investigators had no access to identifiable information.

### Patient population

This study used data from the HOLLAND (HistopathOLogy of Lung Aging aNd COPD) study, which is a comprehensive immunohistochemistry study conducted at the Department of Pathology and Medical Biology of the UMCG. The HOLLAND study aims to investigate differences in markers in lung surgery resection tissues in relation to chronic lung diseases (including COPD) and aging [18, 19]. For the current study, we included 27 patients with COPD and 40 non‐COPD control subjects. The COPD group consisted entirely of exsmokers, while the control group included never‐smokers (*n* = 12), exsmokers (*n* = 18), and current smokers (*n* = 10). Among the 27 COPD patients were 15 patients with mild COPD (GOLD II/III) and 12 patients with severe COPD (GOLD IV).

### Immunohistochemistry

Immunohistochemical staining was performed on the lung tissues derived from patients with COPD and non‐COPD control donors. The specification of the staining with the used antibodies, their dilution, and appropriate buffers, are presented in Table 1. The tissue samples were fixed in formalin and embedded in paraffin. The sections were cut at a thickness of 3 μm and underwent deparaffinization and rehydration. Antigen retrieval was performed by treating the sections with the appropriate buffer for 15 min at cooking temperature in the microwave (HSPA5, NRP1, CTSL, ADAM17, BSG, ITGA5, and ITGB6) or overnight in an 80 °C incubator (Furin and TMPRSS2). The sections were then washed with phosphate‐buffered saline (PBS) and endogenous peroxidase activity was blocked using 0.3% hydrogen peroxidase (H_2_O_2_) for 30 min and washed again with PBS. An additional blocking step with 15 min Avidin and 15 min Biotin was performed for TMPRSS2, ADAM17, and Furin. Next, the sections were incubated for 1 h at room temperature with the primary antibodies diluted in 1% bovine albumin serum (BSA)/PBS. Following washes, secondary and tertiary antibodies or detection reagents were applied for 30 min, diluted in PBS with 1% BSA, and 1% human AB‐serum. Negative controls (i.e. no primary antibody controls) were also included. Positive staining was visualized using Vector® NovaRED® substrate, SK‐4800 (Vector Laboratories, Burlington, Ontario, Canada). The sections were counterstained with haematoxylin, followed by dehydration, mounting, and scanning with a digital slide scanner Hamamatsu Nanozoomer 2.0HT (Hamamatsu Photonics K.K., Hamamatsu, Shizuoka, Japan) using the 40× objective. Digital images were viewed using the Aperio ImageScope v.12.4.3 software (Leica Biosystems, Nussloch, Germany).

**Table 1:** Specification of quantitative immunohistochemical staining for SARS‐CoV‐2 entry‐associated proteins in COPD patients and non‐COPD controls

Protein	Antigen retrieval conditions	Blocking agent	Primary antibody (dilution) and supplier (cat #no)	Secondary and tertiary antibody/reagent)
**Furin**	10 mm/1 mm Tris/HCL (pH 9.0), o/n in 80 °C incubator	Endogenous H_2_O_2_ Avidin Biotin	Mouse anti‐Furin (1:50) Santa‐Cruz Biotechnology, Dallas, TX, USA (sc‐133,142)	RAM‐Bio (1:100) Agilent Dako, Santa Clara, CA, USA (E0413) Streppo 1:300 Agilent Dako (P0397)
**HSPA5**	10 mm Citrate (pH 6.0), MW 15 min	Endogenous H_2_O_2_	Rabbit anti‐HSPA5 (1:400) Sigma Aldrich, St. Louis, MO, USA (Atlas antibodies, Bromma, Sweden) (HPA038845)	GAR‐PO 1:100 Agilent Dako (P0448) RAG‐PO 1:100 Agilent Dako (P0449)
**NRP1**	10 mm/1 mm Tris/HCL (pH 9.0), MW 15 min	Endogenous H_2_O_2_	Rabbit anti‐NRP1 (1:200) Sigma Aldrich (Atlas antibodies) (HPA03278)	GAR‐PO 1:100 Agilent Dako (P0448) RAG‐PO 1:100 Agilent Dako (P0449)
**CTSL**	10 mm/1 mm Tris/HCL (pH 9.0), MW 15 min	Endogenous H_2_O_2_	Rabbit anti‐CTSL (1:200), Sigma Aldrich (Atlas antibodies) (SAB4300959)	GAR‐PO 1:100 Agilent Dako (P0448) RAG‐PO 1:100 Agilent Dako (P0449)
**ADAM17**	10 mm/1 mm Tris/HCL (pH 9.0), MW 15 min	Endogenous H_2_O_2_ Avidin Biotin	Rabbit anti‐ADAM17 (1:50) Sigma Aldrich (Atlas antibodies) (HPA010738)	GAR‐Bio 1:100 Agilent Dako, (E0432) Streppo 1:100 Agilent Dako (P0397)
**BSG**	10 mm Citrate (pH 6.0), MW 15 min	Endogenous H_2_O_2_	Rabbit anti‐BSG (1:625) Sigma Aldrich (Atlas antibodies) (HPA036048)	GAR‐PO 1:100 Agilent Dako (P0448) RAG‐PO 1:100 Agilent Dako (P0449)
**ITGA5**	10 mm Citrate (pH 6.0), MW 15 min	Endogenous H_2_O_2_	Rabbit anti‐ITGA5 (1:50) Sigma Aldrich (Atlas antibodies) (HPA002642)	GAR‐PO 1:100 Agilent Dako (P0448) RAG‐PO 1:100 Agilent Dako (P0449)
**ITGB6**	10 mm/1 mm Tris/HCL (pH 9.0), MW 15 min	Endogenous H_2_O_2_	Rabbit anti‐ITGB6 (1:100) Sigma Aldrich (Atlas antibodies) (HPA023626)	GAR‐PO 1:100 Agilent Dako (P0448) RAG‐PO 1:100 Agilent Dako (P0449)
**TMPRSS2**	10 mm/1 mm Tris/HCL (pH 9.0), o/n in 80°C incubator	Endogenous H_2_O_2_ Avidin Biotin	Rabbit anti‐TMPRSS2 (1:50) Sigma Aldrich (Atlas antibodies) (HPA0335787)	GAR‐Bio 1:100 Agilent Dako (E0432) Streppo 1:100 Agilent Dako (P0397)

Abbreviations: GAR‐bio, goat anti‐rabbit biotinylated; GAR‐PO, goat anti‐rabbit peroxidase; MW, microwave; o/n, overnight; RAG‐PO, rabbit anti‐goat peroxidase; RAM‐Bio, rabbit anti‐mouse biotinylated; Streppo, streptavidin‐peroxidase.

### Image analysis

The bronchial epithelium of the lung was analysed for the airway epithelial expression of proteins in COPD compared to control donors, as described previously [19]. Aperio ImageScope software v.12.4.3 (Leica Biosystems) was used to extract images containing airways from the scans. Depending on the donor, a maximum of 10 airways per scan were extracted. Specific areas of interest (i.e. airway epithelial layer from basement membrane to luminal cell surface) were then captured using Adobe Photoshop software (Adobe Inc., San Jose, CA, USA). Fiji/ImageJ software (National Institutes of Health, Bethesda, MD, USA) [20] was used to quantify the percentage of stained tissue area and the mean intensity of pixels reaching the threshold for positive staining using a colour deconvolution plugin by Landini *et al* [21] and by following the protocol previously described by Ruifrok *et al* [22]. R software v.4.0.0 (R Foundation for Statistical Computing, Vienna, Austria; https://www.R-project.org/, accessed 15 August 2025) was used to calculate the percentage of positively stained tissue and the mean intensity by using the following formulas:

$$\text{Area}\;\left(\%\right)=\frac{\text{Number of pixels positive for NovaRed}}{\text{Number of pixels in total tissue}}✕100$$

$$\text{Mean intensity}=255-\frac{\mathrm{Sum}\;\text{of intensities of pixels positive for NovaRed}}{\text{Total number of pixels positive for NovaRed}}$$

### Statistical analyses

Baseline demographic and clinical characteristics were presented as medians [interquartile ranges] – as these data followed a nonnormal distribution – or proportions *n* with corresponding percentages (%). Normality was assessed using histograms and normal probability plots (P–P plots). Between‐group comparisons were performed using Mann–Whitney *U*‐tests in case of continuous variables, while nominal variables were compared using chi‐square tests. Linear mixed models with a random effect on intercept per subject for airway were used to assess the associations between SARS‐CoV‐2 entry‐associated protein expression in bronchial epithelium and the clinical factors smoking status and COPD. Corrections for age and sex were applied by entering these as fixed effects in the linear mixed models. Finally, correlations between SARS‐CoV‐2 entry‐associated protein expression and FEV_1_%pred as a measure of lung function and an indication of COPD severity were also assessed using linear mixed models. *p* values <0.05 were considered statistically significant. As appropriate, log(natural)‐transformation to the protein expression data was applied. Data were analysed using IBM SPSS Statistics v.28.0 (IBM Corp., Armonk, NY, USA) and visualized using Adobe Illustrator v.29.3 (Adobe Inc., San Jose, CA, USA), as well as the Python programming language v.3.9.7 (Python Software Foundation, Wilmington, DE, USA; URL https://www.python.org/, accessed 15 August 2025), using the *pandas* v.1.3.3 (https://pandas.pydata.org/, accessed 15 August 2025), *numpy* v.1.21.2 (https://numpy.org/, accessed 15 August 2025), *matplotlib* v.3.4.3 (https://matplotlib.org/, accessed 15 August 2025), *seaborn* v.0.11.2 (https://seaborn.pydata.org/, accessed 15 August 2025), and *zepid* v.0.9.1 (https://zenodo.org/records/7242696, accessed 15 August 2025) packages.

## Results

### Study population characteristics

The demographic and clinical characteristics of included COPD patients (*n* = 27) and non‐COPD controls (*n* = 40) are presented in Table 2. No significant differences were observed in age between COPD patients (median [IQR] = 67.0 [52.0–73.0] years) and non‐COPD controls (62.0 [52.3–71.8] years, *p* = 0.664). However, significant differences were found in the female/male ratio between COPD patients (37.0% female) and non‐COPD controls (63.6% female, *p* = 0.024). Lung function was (by definition) significantly lower in COPD patients than in non‐COPD controls, as demonstrated by FEV_1_%pred (51.6 [17.4–65.0] *versus* 102.0 [89.5–113.7], *p* < 0.001) and FEV_1_/FVC% (50.0 [28.7–63.0] *versus* 75.0 [72.0–80.3], *p* < 0.001).

**Table 2:** Demographic and clinical characteristics of COPD patients and non‐COPD controls

	COPD patients (*n* = 27)	Non‐COPD controls (*n* = 40)	*p* value
Age (years), median [IQR]	67.0 [52.0–73.0]	62.0 [52.3–71.8]	0.664^a^
Female sex, *n* (%)	10 (37.0)	26 (65.0)	**0.024** ^b^
Smoking habits, *n* (%)			**<0.001** ^b^
Never‐smoker, *n* (%)	0 (0.0)	12 (30.0)	
Ex‐smoker, *n* (%)	27 (100.0)	18 (45.0)	
Current smoker, *n* (%)	0 (0.0)	10 (25.0)	
FEV_1_ pred (%), median [IQR]	51.6 [17.4–65.0]	102.0 [89.5–113.7]	**<0.001** ^a^
FEV_1_/FVC (%), median [IQR]	50.0 [28.7–63.0]	75.0 [72.0–80.3]	**<0.001** ^a^

*Note*: Data are presented as medians [IQR] or proportions *n* with corresponding percentages (%).

^a^ Mann–Whitney *U*‐test.

^b^ Pearson's chi‐square test. Abbreviations: COPD, chronic obstructive pulmonary disease; FEV_1_, forced expiratory volume in 1 s; FVC, forced vital capacity; IQR, interquartile range.

### Evaluation of SARS‐CoV‐2 entry‐associated protein expression in bronchial epithelium of COPD patients and non‐COPD controls

**Figure 1:**
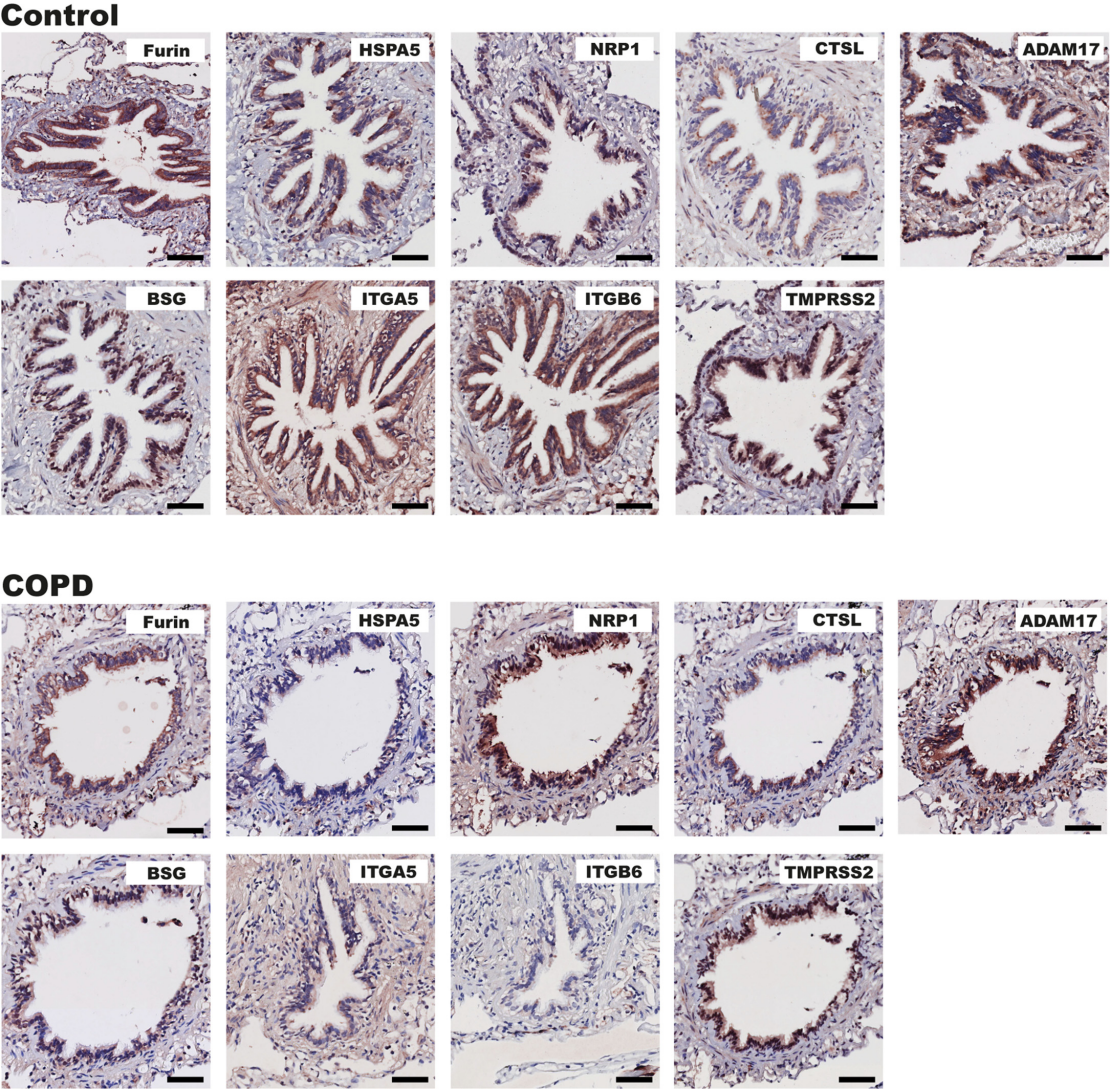
Immunohistochemical staining of SARS‐CoV‐2 entry‐associated proteins in control and COPD airways. Images are representative of protein expression patterns observed in controls (*n* = 40) and COPD (*n* = 27). Original magnification 200×. Scale bar, 60 μm. ADAM17, a disintegrin and metalloprotease 17; BSG, basigin; CTSL, cathepsin L; HSPA5, heat shock protein 5; ITGA5, integrin α5; ITGB6, integrin β6; NRP1, neuropilin 1; TMPRSS2, transmembrane protease serine 2.

**Figure 2:**
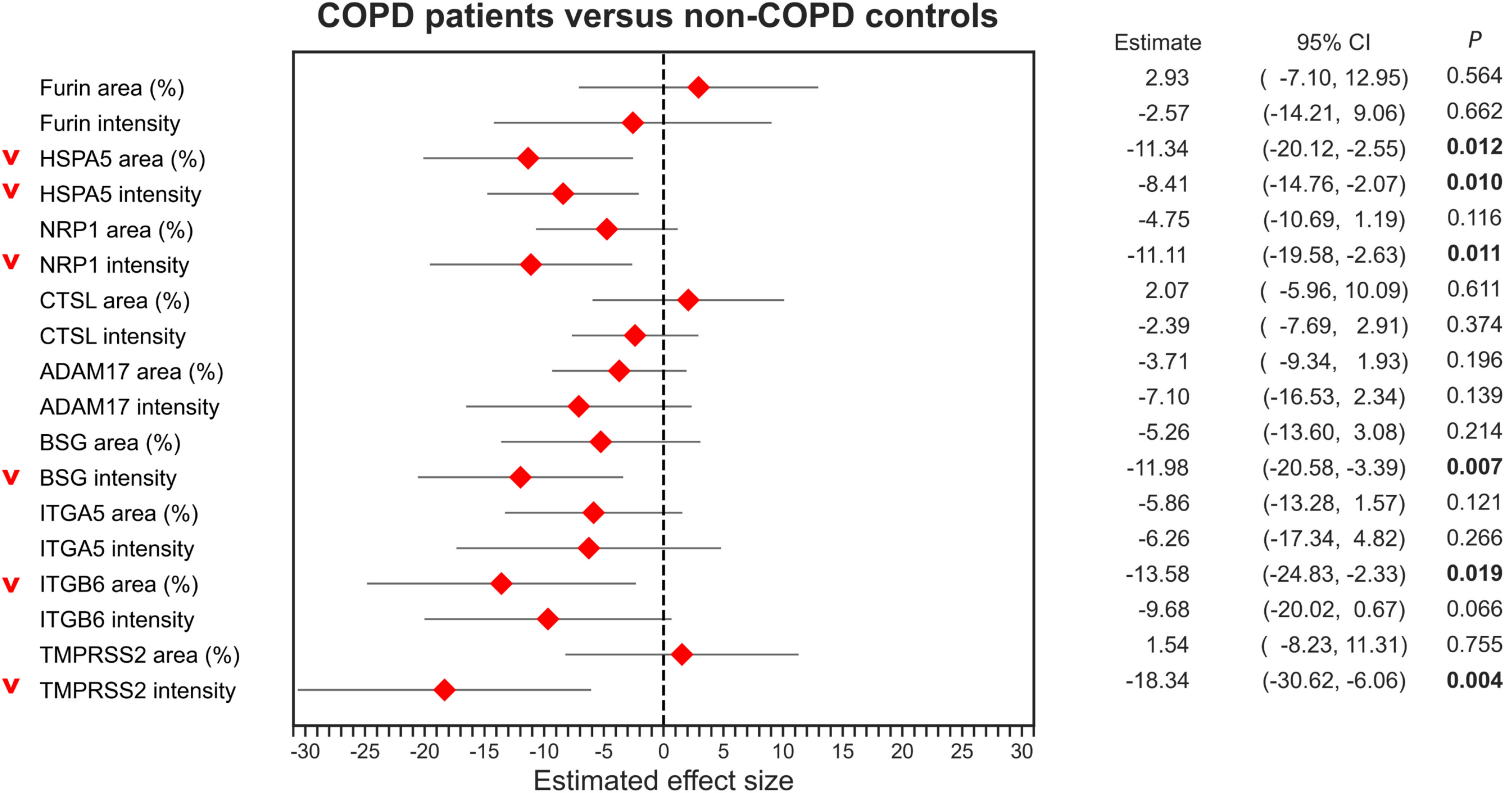
Forest plots demonstrating estimates of fixed effects with corresponding 95% confidence intervals (CI) for differences in SARS‐CoV‐2 entry‐associated protein airway expressions between exsmoking COPD and exsmoking non‐COPD controls. ADAM17, a disintegrin and metalloprotease 17; BSG, basigin; CTSL, cathepsin L; HSPA5, heat shock protein 5; ITGA5, integrin α5; ITGB6, integrin β6; NRP1, neuropilin 1; TMPRSS2, transmembrane protease serine 2. Bold *p* values indicate statistical significance (*p* < 0.05).

The bronchial epithelium of exsmoking COPD patients and non‐COPD controls was analysed for the percentage area and mean intensity of the above‐described SARS‐CoV‐2 entry‐associated protein expressions. Visualization of the staining patterns in COPD and non‐COPD lung tissue is shown in Figure 1, and the differences in entry factor protein expression between COPD and non‐COPD control are shown in Figure 2. COPD showed lower HSPA5 percentage area (*p* = 0.012) and mean intensity (*p* = 0.010). In addition, lower mean intensity of NRP1 (*p* = 0.011), BSG (*p* = 0.007), and TMPRSS2 (*p* = 0.004) as well as lower percentage area of positive ITGB6 staining (*p* = 0.019) were observed in COPD. No significant differences were observed in the airway expressions of Furin, CTSL, ADAM17, and ITGA5. Finally, in COPD patients only, correlations between the protein expression patterns of interest and lung function (FEV_1_%pred measurements) were evaluated, showing a significant negative association with the percentage area of BSG (*p* = 0.013) (supplementary material, Figure S1). To summarize, we observed an overall lower expression of SARS‐CoV‐2 entry factors in the bronchial epithelium of COPD patients.

### Evaluation of smoking‐associated protein expression in bronchial epithelium of non‐COPD controls

**Figure 3:**
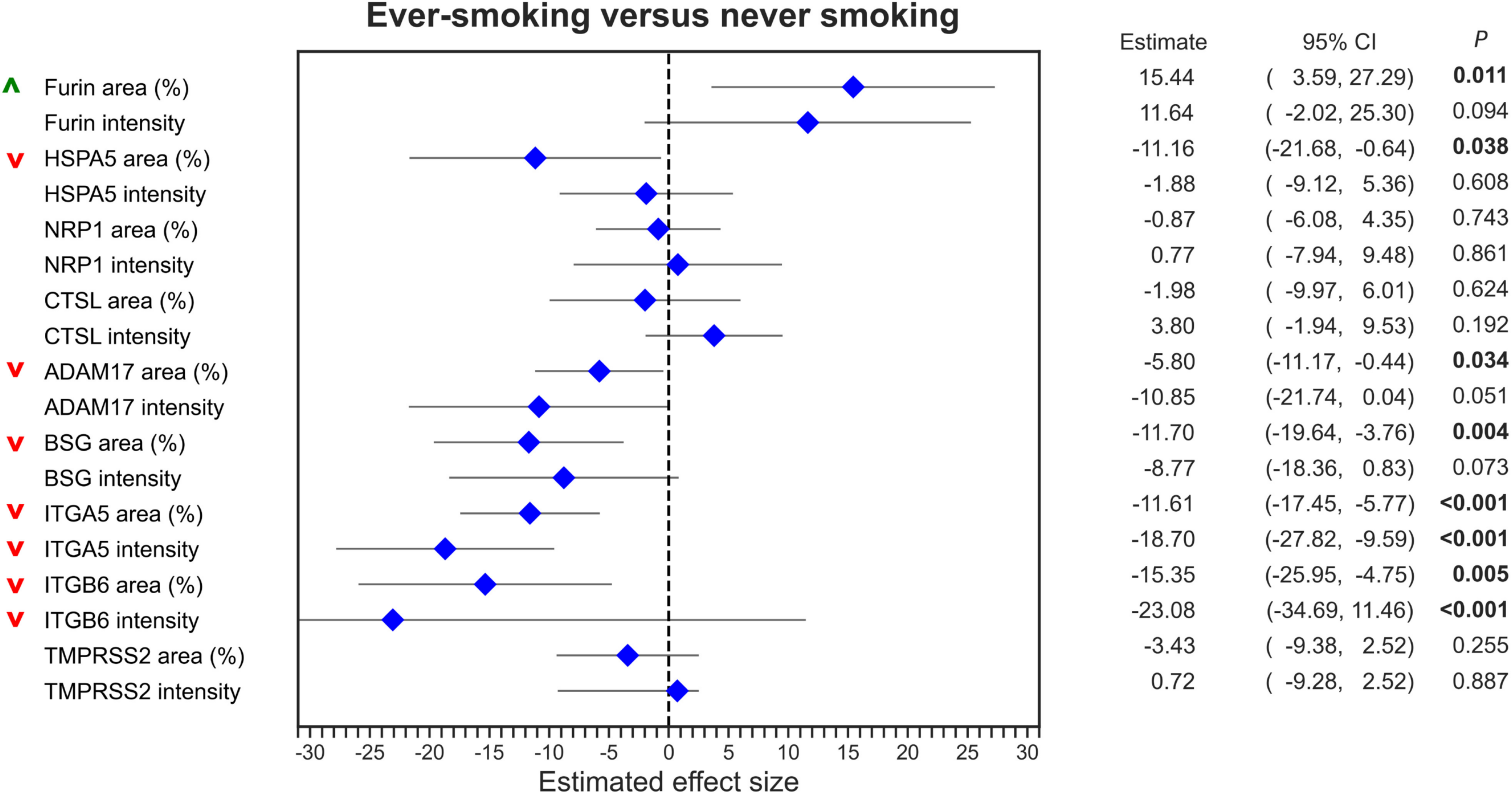
Forest plots demonstrating estimates of fixed effects with corresponding 95% confidence intervals (CI) for differences in SARS‐CoV‐2 entry‐associated protein expressions in airways of ever‐smokers compared to never‐smokers. ADAM17, a disintegrin and metalloprotease 17; BSG, basigin; CTSL, cathepsin L; HSPA5, heat shock protein 5; ITGA5, integrin α5; ITGB6, integrin β6; NRP1, neuropilin 1; TMPRSS2, transmembrane protease serine 2. Bold *p* values indicate statistical significance (*p* < 0.05).

Next, we established the effect of smoking status on the protein expression of SARS‐CoV‐2 entry factors in the bronchial epithelium in non‐COPD controls. We observed an age‐ and sex‐independent higher percentage area of positive Furin staining in ever‐smokers (*p* = 0.011), as well as a lower percentage area of HSPA5 (*p* = 0.038), ADAM17 (*p* = 0.034), and BSG (*p* = 0.004) staining (Figure 3). In addition, ever‐smokers showed both lower expressions of the percentage area and mean intensity of integrins ITGA5 (*p* < 0.001) and ITGB6 (*p* = 0.005 and *p* < 0.001, respectively). A lower percentage area of HSPA5 and ITGB6 were also observed in COPD (see above), indicating lower airway expressions with smoking, which was even more pronounced in COPD (supplementary material, Figure S2). When comparing the staining of SARS‐CoV‐2 entry‐associated protein expression between exsmokers and current smokers (excluding never‐smokers), only a lower mean intensity of ITGA5 was observed in current smokers than in exsmokers (*p* = 0.020) (supplementary material, Figure S3). To summarize, ever‐smoking was associated with a higher protein expression of Furin and lower protein expression of HSPA5, ADAM17, BSG, ITGA5, and ITGB6, indicating an overall lower airway expression of SARS‐CoV‐2 entry factors with smoking.

## Discussion

In this study we investigated the expression of SARS‐CoV‐2 entry‐associated proteins in the bronchial epithelium of COPD patients, as this may provide a potential mechanism underlying COVID‐19 susceptibility in this patient population. Interestingly, our results indicated different airway expression patterns of most SARS‐CoV‐2 entry‐associated proteins in COPD patients as compared to non‐COPD controls, with significantly lower protein expression of HSPA5 (percentage area and mean intensity), ITGB6 (percentage area), as well as NRP1, BSG, and TMPRSS2 (mean intensity). No significant differences were observed for Furin, CTSL, ADAM17, and ITGA5. Although the percentage area of BSG was similar in COPD and controls, within the COPD group, there was a negative association with FEV_1_%pred as measured for lung function (although limited in effect), indicating slightly higher BSG expression in more severe COPD. In non‐COPD controls, we showed smoking‐associated differences for Furin, HSPA5, ADAM17, BSG, ITGA5, and ITGB6, with mostly lower airway expression (except for Furin) in ever‐smokers compared to never‐smokers. Our results are important, as they provide novel and granular insight on the protein level into involvement of the bronchial epithelial expression patterns of alternative factors in SARS‐CoV‐2 entry.

Although there is mounting evidence for the propensity of COPD patients to progress to severe illness in COVID‐19, their susceptibility to acquiring SARS‐CoV‐2 infection remains unclear [9]. Similar susceptibility as in non‐COPD has been suggested, as COPD patients seemed not to be overrepresented in COVID‐19. For example, an early large epidemiological study of all diagnosed COVID‐19 cases in PR China at the beginning of 2020 found a COPD prevalence of 2.4% (*n* = 511) [23]. A similar pooled prevalence of 2% was observed in two meta‐analyses of 2,473 and 6,261 confirmed COVID‐19 cases, conducted in 2020 and 2021, respectively [24, 25]. It must be mentioned, however, that the included studies concerned a hospitalized population, possibly creating a selection bias toward more severe illness. Another meta‐analysis from 2022 reported the country‐specific COPD prevalence in the COVID‐19 population (mostly hospital‐based) *versus* in the general population, showing varying results with significantly higher COPD prevalence rates in COVID‐19 in Denmark, the USA, Italy, and The Netherlands, and a statistically lower prevalence in Canada and PR China [6]. However, significant heterogeneity between the studies, together with unaccounted differences between the COVID‐19 and general populations (such as age and sex) may have influenced the results. Furthermore, given the concern that COPD increases COVID‐19 risk, behavioural changes with earlier and stricter social distancing adherence may have affected COVID‐19 susceptibility due to reduced exposure [26, 27]. As a result, the true biological susceptibly might be underestimated due to these protective behaviours.

SARS‐CoV‐2 uses ACE2 as a cell receptor to enter the host cell, which in the nose and airways is primarily expressed in secretory goblet cells and in alveolar type 2 cells [28, 29]. The tissue distribution of ACE2 does not directly correlate with organ involvement in SARS‐CoV‐2 infection, with a relatively low expression of ACE2 in lung tissue, which has been one of the reasons to hypothesize the involvement of alternative SARS‐CoV‐2 entry factors [30, 31]. It must be mentioned, however, that the large surface area of alveolar epithelial cells may explain, in part, the lung's vulnerability to SARS‐CoV‐2‐induced pathology despite relatively low ACE2 expression. Nevertheless, ACE2 – albeit essential – has been demonstrated not to be the only factor determining SARS‐CoV‐2 entry, as the coexpression of other cellular receptors (including BSG, NRP1, HSPA5, and integrins) and priming proteases (including TMPRRS2, CTSL, and Furin) are also important alternative components of SARS‐CoV‐2 infection [10, 12, 13, 14, 15, 16, 17]. That study focused on those SARS‐CoV‐2 entry factors (other than ACE2) to take into account different viral entry pathways and, thereby, seek an elaborate understanding of SARS‐CoV‐2 susceptibility.

Our study demonstrates that, at a protein level, most of the studied SARS‐CoV‐2 entry‐associated proteins showed lower expression in airway epithelium of COPD patients compared to non‐COPD controls, which was significant for HSPA5, NRP1, BSG, TMPRSS2, and ITGB6. This suggests a potential less cellular availability for SARS‐CoV‐2 entry, theoretically lowering susceptibility to infection, that may seem to contradict the existing literature that does not conclusively show that COPD patients are less likely to acquire COVID‐19. While the airway expression of most SARS‐CoV‐2 entry factors may be lower, it is also interesting to focus on those proteins that remained unchanged between COPD and controls, including Furin, CTSL, ADAM17, and ITGA5, as these factors could still facilitate infection. In COPD, the microenvironment of the airways, characterized by increased mucus production of a more sticky nature and impaired ciliary function, may hinder clearance of pathogens, making COPD patients more susceptible for respiratory infections in general and, consequently, more prone to exacerbations [32]. The prolonged exposure to SARS‐CoV‐2 in the airways could result in longer contact between the virus and the epithelium, implying that a lower or similar airway epithelial expression of SARS‐CoV‐2 entry factors may still be sufficient for infection. Thus, it could be speculated that less viral entry expression would be required for similar—or even higher—SARS‐CoV‐2 susceptibility in COPD lungs. Furthermore, lower expression of SARS‐CoV‐2 entry factors in COPD airways potentially allows more intact virus to reach the alveoli, characterized by relatively low airflow velocity and a large surface area. It can be speculated that this increases the likelihood of parenchymal involvement after initial SARS‐CoV‐2 infection, possibly explaining the more severe disease course observed in COPD, although this theory warrants further investigation.

To our knowledge, previous studies investigating preinfection expression patterns of SARS‐CoV‐2 entry‐related factors in COPD lung tissue on both a gene level [33, 34, 35, 36] and protein level [36, 37] demonstrated variable results. For example, a large study performing transcriptomic analysis on single‐cell RNA‐sequencing data showed comparable expression profiles of SARS‐CoV‐2 entry factors (including ACE2, TMPRSS2, CTSL, and Furin) in the epithelial cell population of lung samples from healthy and chronic lung disease patients, including COPD patients [34]. Immunohistochemical studies in COPD patients are limited, while they are important, as gene expression patterns may not necessarily be the same as its protein expression patterns. Fließer *et al* investigated the expression levels of ACE2 and other cofactors on both a gene and protein level in lung tissue samples, demonstrating upregulation of ACE2 and TMPRSS2 expression in both bronchial and alveolar epithelial cells, whereas Furin and BSG were downregulated in COPD patients, as compared to controls [36]. As some of the studied proteins are involved in activation of proinflammatory pathways and tissue remodelling, one would expect higher expressions in COPD. For example, ADAM17 – a key sheddase that cleaves various cell surface proteins – may modulate inflammation in COPD pathogenesis by cleaving key cytokines such as tumour necrosis factor alpha (TNF‐α) and interleukin‐6‐receptor (IL‐6R), thereby enhancing proinflammatory signalling and promoting lung cell damage [38, 39]. Furthermore, integrins are thought to play a role in COPD through activation of transforming growth factor beta (TGF‐β), with a well‐established role in fibroinflammatory processes [40, 41]. Nevertheless, the interplay of these factors in COPD is complex, and continuous inflammatory stress could potentially result in compensatory responses that downregulate certain pathways to protect lung cells from further damage. Furthermore, other (noninflammatory) factors in COPD could potentially explain reduced entry factor expression, as has been observed for inhaled corticosteroids [42], which may also apply to smoking (see below).

COPD has been established as a significant risk factor for adverse clinical outcomes in COVID‐19 [1, 2, 3, 4, 5, 6]. The exact mechanisms underlying the altered propensity to severe COVID‐19 are complex and currently poorly understood, but some mechanisms can be suggested. COPD patients are generally older, with an increased incidence of comorbidities and smoking history [5, 6]. Furthermore, COPD has been associated with weakened immune responses, with impaired immunity to respiratory infection in general [43], and prolonged SARS‐CoV‐2 shedding [44]. COPD patients with already poor lung function may be more vulnerable to COVID‐19‐related pulmonary events – including acute respiratory distress syndrome (ARDS), thromboembolism, and secondary superinfection – thereby reducing the threshold for respiratory failure. In addition, inhaled corticosteroids are commonly used in COPD, which has been suggested to impact COVID‐19 outcomes – either by exposing patients to an increased risk due to suppression of interferon‐mediated immunity (and thereby an impaired antiviral response), or by protection against a hyperinflammatory response often observed in severe disease [45]. Differences in the expression of SARS‐CoV‐2 entry‐associated proteins could potentially contribute to COVID‐19 severity to some extent by facilitating faster spread of SARS‐CoV‐2 into the lungs. However, it must be stressed that the current study exclusively focused on airway expression profiles of SARS‐CoV‐2 entry‐associated factors prior to SARS‐CoV‐2 infection, as the samples were collected before the pandemic started. Once infection has become established and initial cellular injury has taken place, it is unclear whether and in which way preinfection viral entry expression patterns contribute to the propagation of lung injury, and thereby determine progression to severe COVID‐19. Instead, SARS‐CoV‐2 infection itself may induce changes in these viral entry‐associated factors by upregulation or downregulation, which may differ between COPD and healthy lungs, and could potentially influence COVID‐19 pathogenesis [46]. A previous study investigated the changes in SARS‐CoV‐2 coreceptor expression using SARS‐CoV‐2 inoculated primary bronchial epithelial cells (pBECs), demonstrating higher susceptibility to SARS‐CoV‐2 infection in COPD pBECs, with upregulation of TMPRSS2 and cathepsin B (CTSB) upon infection, as well as elevated baseline expression of CTSL in COPD donors [47]. As our study relied solely on a preinfection airway expression pattern, the focus of our research was primarily on the susceptibility to acquiring COVID‐19 rather than on progression to severe disease.

Smoking induces chronic lung inflammation and is considered a key risk factor in the pathogenesis of COPD. Indeed, all the COPD participants in our study had a history of smoking. Current smoking is an established risk factor for COVID‐19 [48], for which higher ACE2 expression has been attributed as a partial explanation in previous studies [48, 49, 50, 51]. Smoking‐associated differences in the expression of other SARS‐CoV‐2 entry‐associated factors have been investigated in the literature, with varying results. For example, a study using bronchoscopically isolated tissue demonstrated that current smoking was associated with higher gene expression of ACE2, TMPRRS2, Furin, and BSG in bronchial brushings, whereas a negative association was found with CTSL [50]. Cai *et al* demonstrated that gene expressions of ACE2 and Furin – but not TMPRRS2 – were upregulated in ever‐smokers compared to nonsmokers [51]. In our study, investigating protein expression in non‐COPD controls only, we showed smoking‐associated differences for Furin, HSPA5, ADAM17, BSG, ITGA5, and ITGB6, with lower airway expression profiles (except for Furin) in ever‐smokers compared to never‐smokers. Our findings suggest that smoking is associated with generally lower airway expressions of SARS‐CoV‐2 entry‐associated proteins, which may be even more pronounced in COPD patients. Furthermore, while integrins (such as ITGA5 and ITGB6) have previously been indicated to facilitate SARS‐CoV‐2 cell entry, our results suggest that the integrin pathway is not the primary alternative entry mechanism in our cohort [14, 15, 16, 17].

In this study, extensive immunohistochemical analyses were performed to assess SARS‐CoV‐2 entry‐associated protein expression in the bronchial epithelium of COPD patients and non‐COPD controls, while correcting for relevant clinical confounding factors. Our sample size, with an assessment of up to 10 airways per individual, provided sufficient data for proper analysis. Furthermore, analysing such a wide range of SARS‐CoV‐2 entry‐associated proteins makes this study unique and allows for extensive appreciation of SARS‐CoV‐2 infection and susceptibility. The observed differences in percentage area and mean intensity of the positive staining provided us with different information on the distribution and amount of protein expression within the bronchial epithelium. At the same time, however, there are some limitations to this study that warrant recognition.

First, we solely investigated SARS‐CoV‐2 entry‐associated protein expression in the bronchial epithelium, whereas other compartments – including lung parenchyma – would also be relevant to SARS‐CoV‐2 susceptibility. Further work will be required to gain a more granular insight into the differences in expression patterns between COPD patients and controls in other compartments of the lung. Furthermore, our study was conducted in lung tissue that was not exposed to SARS‐CoV‐2, whereas SARS‐CoV‐2 infection itself may induce changes in viral entry factor expression that could potentially affect COVID‐19 pathophysiology. This approach also prevented us from drawing direct associations between our findings and COVID‐19 status or clinical outcomes. While we have discussed potential interpretations of our findings, we realize that the exact underlying mechanisms and their consequences on COVID‐19 susceptibility remain to be determined, and alternative explanations may exist. Nevertheless, despite these limitations, this study offers a thorough evaluation of alternative SARS‐CoV‐2 entry factors in COPD and smokers, shedding light on the potential mechanisms behind COVID‐19 susceptibility.

## Conclusion

This study provides an in‐depth assessment of selected alternative SARS‐CoV‐2 entry‐associated protein expression in the bronchial epithelium of COPD patients, demonstrating generally lower expression of these proteins as compared to non‐COPD controls, implying a lower cellular availability for SARS‐CoV‐2 entry. However, the presence of similar airway epithelial expression of certain proteins, combined with the unique microenvironment in COPD with impaired airway clearance, may still facilitate infection due to prolonged exposure in the alveolar compartment also on a very large surface. Our findings offer potential mechanistic insights into SARS‐CoV‐2 entry dynamics in the bronchial epithelium of COPD patients, enhancing our understanding of COVID‐19 susceptibility in this patient population.

## Author contributions statement

C‐AB and WT contributed to conceptualization and provided supervision. JB and MRL conceived and carried out experiments. LEVvE and ZS performed data analysis. LEVvE, ZS, C‐AB and WT interpreted the data. WT acquired funding for the study. LEVvE and ZS prepared the initial draft, while all authors reviewed and edited the final article. All authors approved the final submitted version.

## Supporting information


**Figure S1.** Forest plots demonstrating estimates of fixed effects with corresponding 95% confidence intervals (CI) for associations between SARS‐CoV‐2 entry‐associated protein expressions and FEV1%pred in COPD patients
**Figure S2**. Scatter plots demonstrating lower expressions of HSPA5 area% and ITGB6 area% in ever smokers, which are even further decreased in COPD patients
**Figure S3**. Forest plots demonstrating estimates of fixed effects with corresponding 95% confidence intervals (CI) for associations between SARS‐CoV‐2 entry‐associated protein expressions and current smoking

## Data Availability

The data that support the findings of this study are available from the corresponding author, (LEVvE), upon reasonable request.
