# Supplementary material for: Expression of SARS‐CoV‐2 entry‐associated proteins in COPD airways: an immunohistochemical study
Source: J Pathol. 2025 Oct 6;267(4):424–34. doi: 10.1002/path.6477 (PMC12596910; doi:10.1002/path.6477)
Supplement: Supplementary file 1 — Figure S1. Forest plots demonstrating estimates of fixed effects with corresponding 95% confidence intervals (CI) for associations between SARS‐CoV‐2 entry‐associated protein expressions and FEV1%pred in COPD patients Figure S2. Scatter plots demonstrating lower expressions of HSPA5 area% and ITGB6 area% in ever smokers, which are even further decreased in COPD patients Figure S3. Forest plots demonstrating estimates of fixed effects with corresponding 95% confidence intervals (CI) for associations between SARS‐CoV‐2 entry‐associated protein expressions and current smoking [file PATH-267-424-s001.docx]

**Expression of SARS-CoV-2 entry-associated proteins in COPD airways: an immunohistochemical study**

LE Vlaming-van Eijk *et al. J Pathol* <https://doi.org/10.1002/path.6477>

**Supplementary Figures S1–S3**

**
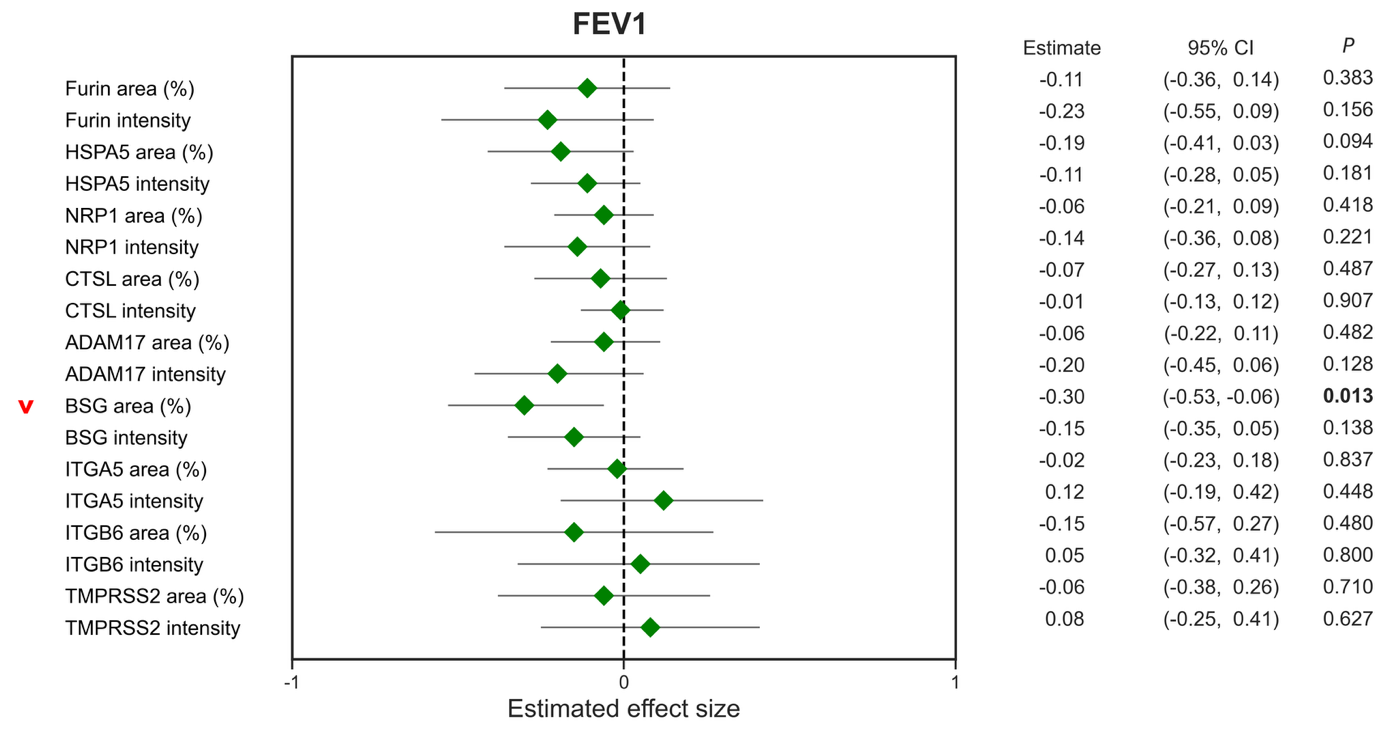
**

**Figure S1. Forest plots demonstrating estimates of fixed effects with corresponding 95% confidence intervals (CI) for associations between SARS-CoV-2 entry-associated protein expressions and FEV_1_%pred in COPD patients.** Abbreviations: ADAM17, a disintegrin and metalloprotease 17; BSG, basigin; CTSL, cathepsin L; HSPA5, heat shock protein 5; ITGA5, integrin alpha-5; ITGB6, integrin beta-6; NRP1, neuropilin 1; TMPRSS2, transmembrane protease serine 2. The **Bold** *p*-value indicates statistical significance (< 0.05).


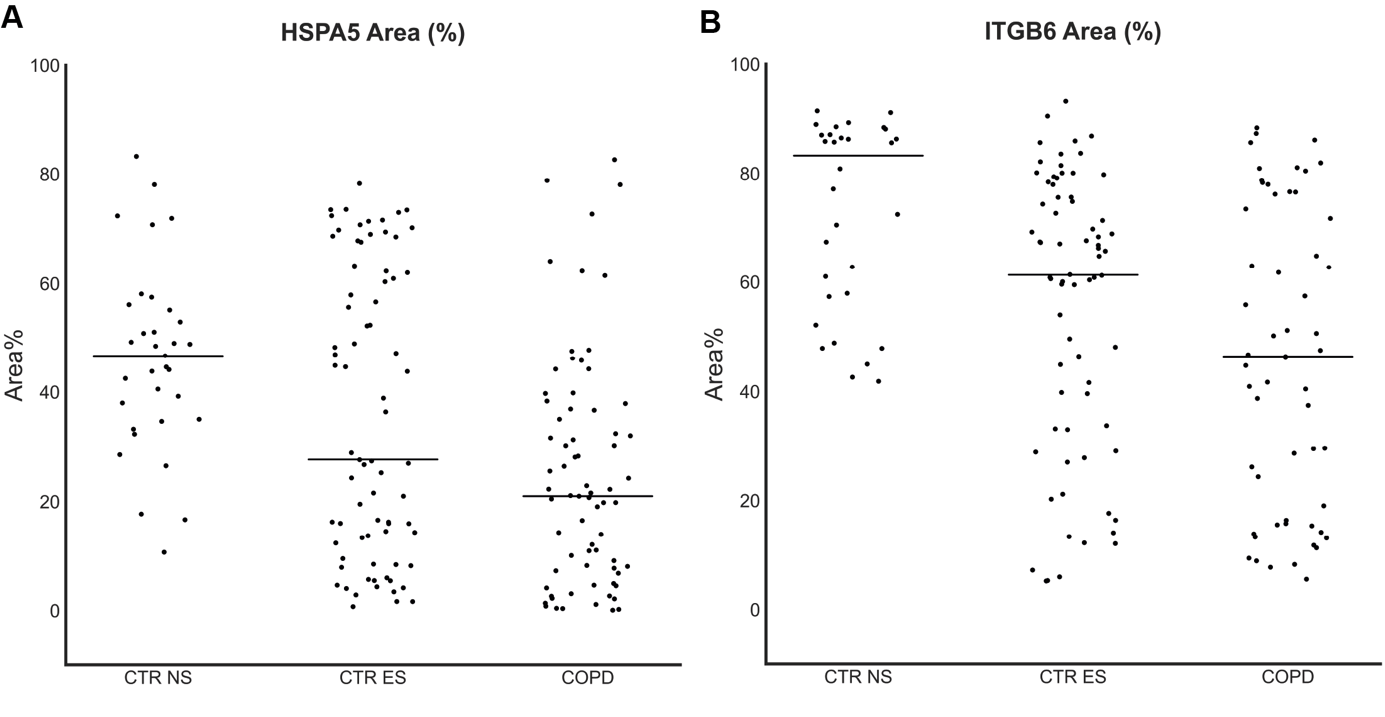


**Figure S2. Scatterplots demonstrating lower expressions of HSPA5 area% and ITGB6 area% in ever-smokers, which are even further decreased in COPD patients.** Abbreviations: CTR ES, control ever-smoker; CTR NS, control never smoker; HSPA5, heat shock protein 5; ITGB6, integrin β6.


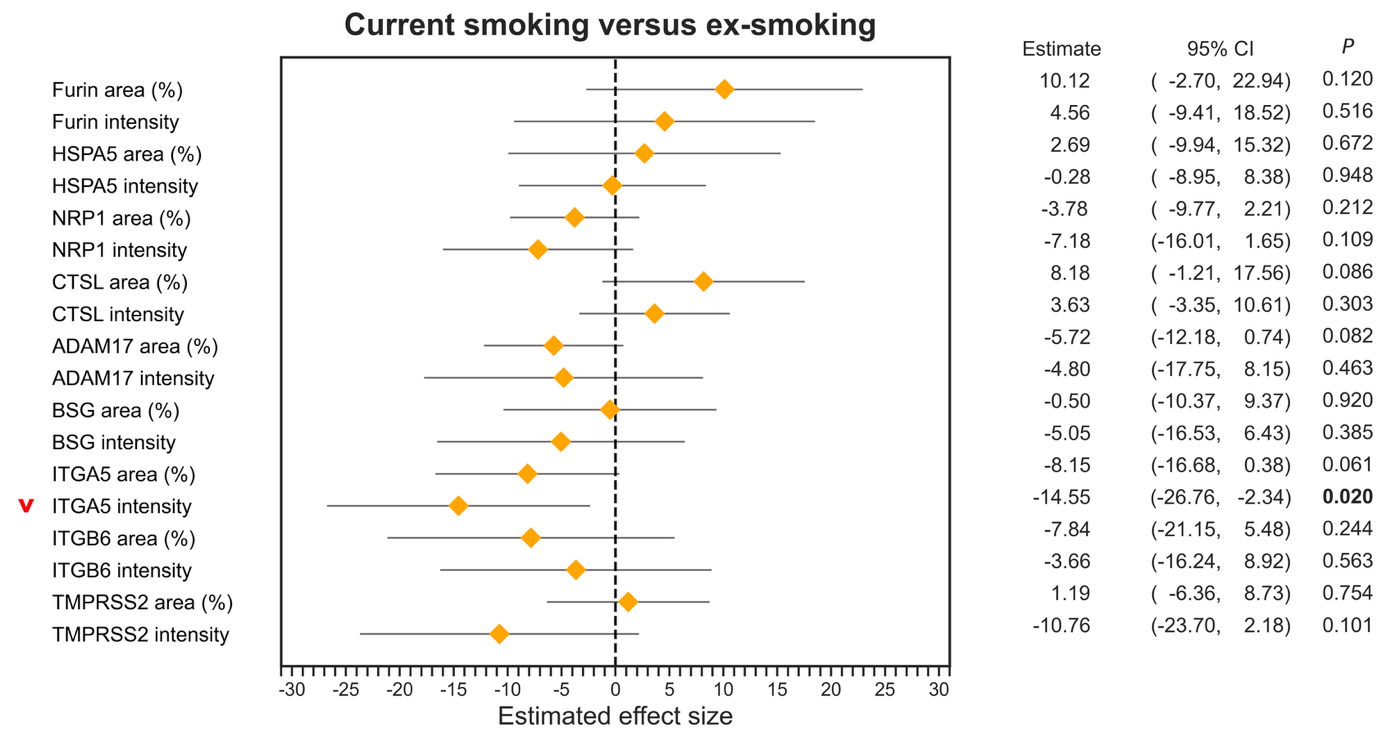


**Figure S3**. **Forest plots demonstrating estimates of fixed effects with corresponding 95% confidence intervals (CI) for associations between SARS-CoV-2 entry-associated protein expressions and current smoking.** Abbreviations: ADAM17, a disintegrin and metalloprotease 17; BSG, basigin; CTSL, cathepsin L; HSPA5, heat shock protein 5; ITGA5, integrin alpha-5; ITGB6, integrin beta-6; NRP1, neuropilin 1; TMPRSS2, transmembrane protease serine 2. The **bold** *p*-value indicates statistical significance (< 0.05).
